# A Semi-Supervised Reduced-Space Method for Hyperspectral Imaging Segmentation

**DOI:** 10.3390/jimaging7120267

**Published:** 2021-12-07

**Authors:** Giacomo Aletti, Alessandro Benfenati, Giovanni Naldi

**Affiliations:** Environmental Science and Policy Department, Università degli Studi di Milano, 20133 Milan, Italy; giacomo.aletti@unimi.it (G.A.); giovanni.naldi@unimi.it (G.N.)

**Keywords:** hyperspectral image segmentation, linear discriminant analysis, spectral similarity, random walks

## Abstract

The development of the hyperspectral remote sensor technology allows the acquisition of images with a very detailed spectral information for each pixel. Because of this, hyperspectral images (HSI) potentially possess larger capabilities in solving many scientific and practical problems in agriculture, biomedical, ecological, geological, hydrological studies. However, their analysis requires developing specialized and fast algorithms for data processing, due the high dimensionality of the data. In this work, we propose a new semi-supervised method for multilabel segmentation of HSI that combines a suitable linear discriminant analysis, a similarity index to compare different spectra, and a random walk based model with a direct label assignment. The user-marked regions are used for the projection of the original high-dimensional feature space to a lower dimensional space, such that the class separation is maximized. This allows to retain in an automatic way the most informative features, lightening the successive computational burden. The part of the random walk is related to a combinatorial Dirichlet problem involving a weighted graph, where the nodes are the projected pixel of the original HSI, and the positive weights depend on the distances between these nodes. We then assign to each pixel of the original image a probability quantifying the likelihood that the pixel (node) belongs to some subregion. The computation of the spectral distance involves both the coordinates in a features space of a pixel and of its neighbors. The final segmentation process is therefore reduced to a suitable optimization problem coupling the probabilities from the random walker computation, and the similarity with respect the initially labeled pixels. We discuss the properties of the new method with experimental results carried on benchmark images.

## 1. Introduction

Hyperspectral imaging systems have gained a great amount of attention from researchers in the past few years. The sensors of these systems allow the simultaneous acquisition of hundreds of spectral wavelengths for each image pixel. This detailed spectral information increases the possibility of more accurately discriminating objects, materials, or regions of interest. Furthermore, the fine spatial resolution of the sensors enables the analysis of small spatial structures in the image. The main property of the Hyperspectral images is the strong resolving power for fine spectra, then they have a wide range of applications in agriculture [1,2], food industry [3], geosciences [4,5], biomedical applications [6,7], document image processing [8], environment [9,10], and others. However, the analysis of HSI requires developing specialized methods and algorithms for data processing [11,12,13,14]. The main methods for the processing of the hyperspectral remote sensing images include image correction [11], noise reduction [15], dimensionality reduction [12], and classification [16,17,18,19].

In principle, the spectral information from the available hundreds of narrow bands collected by hyperspectral sensors can help discriminate among spectrally similar object pixels. Then, the accurate discrimination of different regions in the image is possible and the hyperspectral image classification is one of the most active part of the research in the hyperspectral field [18,20,21,22]. However, the HSI technology still faces a series of challenges, mainly including the following problems that need to be solved. The high dimensionality of the hyperspectral data, for example, the spectral reflectance values of hyperspectral images collected by airborne or space-borne imaging spectrometers, is up to hundreds of dimensions. Moreover, factors such as sensors, atmospheric conditions, surrounding environment, and composition and distribution of ground features affect the spatial variability of spectral information. The interference of noise (e.g., Poisson noise [23,24]) and background factors also seriously degrades the quality of the collected data and the corresponding classification accuracy of HSI. Finally, in practical applications, it is extremely difficult to obtain labeled samples to be used in the classification work of a hyperspectral image. The intrinsic properties of hyperspectral images need to be addressed specifically because conventional classification algorithms made for multispectral images do not adapt well to the analysis of hyperspectral images [25].

Several approaches have been proposed for classification of HSI. A subclass of classifiers is based on probabilistic approaches by using statistical tools to find the best class for a given pixel providing a probability of the pixel being a member of each of the possible classes. For instance, the multinomial logistic regression (MLR) classifier [26] supplies a degree of plausibility for such classes. In the sparse version of MLR a Laplacian prior to enforce sparsity is adopted which leads, with some computational limitations, to good generalization capabilities in HSI classification. More recently, an improved version of this classifier has also been proposed [27] using a subspace based method. The idea of applying subspace projection approach relies on the assumption that the samples within each class can approximately lie in a lower-dimensional subspace.

Due to their successful application in several problems of pattern recognition neural networks have also attracted many researchers in the field of the classification of hyperspectral images [28,29,30,31]. The main advantage of these approaches comes from the fact that neural networks do not need prior knowledge about the statistical distribution of the classes. They need the availability of feasible training techniques for nonlinearly separable data and their use has been affected by their training complexity as well as by the number of parameters that need to be tuned. Several neural network-based classification methods have been proposed in the literature that consider both supervised and unsupervised nonparametric approaches [32,33]. Recently, the extreme learning machine (ELM) algorithm has been successfully applied as nonlinear classifiers for hyperspectral data [34,35], and have shown remarkable efficiency in terms of accuracy and computational complexity. Some deep models have also been proposed for hyperspectral data feature extraction and classification [16]. The architecture design is the crucial part of a successful deep learning model together with the availability of an appropriate broad training set.

Another example of a supervised classification approach is support vector machines (SVMs). They have been widely used for the classification of hyperspectral data due to of their ability to handle high-dimensional data with a limited number of training samples [36]. To generalize the SVM for nonlinear classification problems, the so called kernel trick was introduced [37]. However, the sensitivity to the choice of the regularization parameters and the kernel parameters is the most important disadvantage of a kernel SVM. Other methods for HSI classification involve decision trees [38,39], random forests [40], and sparse representation classifiers with dictionary based generative models [41,42].

In this work we present a novel local/global method for semiautomatic multilabel classification of HSI. Semisupervised methods rely on limited information on the objects to recognize inside the data. Graph Clustering CNNs [43] consists of a two-stage clustering strategy in order to reduce the burden of graph convolution computation. The authors in [44] apply a coupled spatial-spectral approach for approximating the convolution on graphs. Autoencoder models are widely employed for HSI classification tasks (Ref. [45] and references therein). As a first step, we consider a preprocessing process using novel feature selection approaches in order to address the curse of dimensionality and reduce the redundancy of high-dimensional data. We adopt a linear discriminant analysis based on the labeled regions in order to project the high dimensional feature space to a lower dimensional subspace. Then we development a similarity index/distance between pixel reduced features of pixel using reduced features of pixel and involving pixels in a neighborhood. We point out that the so-called pixel based or spectral classifier only treats the HSI data as a list of spectral measurement without considering spatial relations of adjacent pixels, thus discarding important information. In a real image, neighboring pixels are related or correlated, both because imaging sensors acquire significant amount of energy from adjacent pixels and because homogeneous structures which are generally large compared to the size of a pixel frequently occurred in the image scene. This spatial contextual information should help for an accurate scene interpretation. Therefore, in order to improve classification results, spectral-spatial classification methods must be developed, which assign each image pixel to one class based on its own spectral values (the spectral information) and information extracted from its neighborhood (the spatial information). Then, the new similarity index includes the contextual spatial information provided in the HSI data considering features of adjacent pixels together. The same strategy was recently consider in [46] for the segmentation of color images.

Using the new similarity index we represent the image with an undirected graph where the set of vertices is the set of pixels present in the image, and the set of the edges consist of the pairs of neighboring pixels in the image. The weight of an edge can be represented by a function based on the difference between the features of each pixel. The vertices set can be partitioned into two sets: the “labeled vertices”, and the rest of the image pixels. In order to label the last vertices we develop a random walker approach which improves the quality of the image segmentation and involves only the minimization of a quadratic function. We remark that our method considers in a different nonlinear way two terms and it is not a simple thresholding step. In fact, the distances between features affect at the same time the similarity between labeled and unlabeled pixels and the construction of the graph for the random walk. We point out that we could consider the random walk method as a Laplacian-based manifold method with a solid theoretical background [47].

The remaining sections of the paper are organized as follows. Section 2 introduces the new method and the improved random walker segmentation algorithm. Moreover, we will also discuss a new definition of non-local distance between the features of the pixels. Section 3 is devoted to the numerical experiments. In this section the proposed method is tested on some benchmark images, in order to evaluate its performance. Following the findings of the case study, the conclusions are presented in the last Section 4 with a discussion about the properties and the possible developments of the approach.

## 2. A Spatial-Spectral Classifier Method for Hyperspectral Images

The detailed spectral information collected by the available hyperspectral sensors improves the capability of discriminating between different objects/sub-regions in an image by providing a division into classes with increased accuracy. This accurate capacity for discrimination makes hyperspectral data a valuable source of information to be fed to advanced classifiers. The output of the classification step is generally known as the classification map.

However, several particular problems should be considered in the classification task of hyperspectral data, among which are the following: the spatial variability of the spectral characteristics, the high number of spectral channels, the high cost of true sample labeling. In this paper we introduce a novel classification method in the semi-automatic approach framework. The general goal is to segment an image into two or more separate regions, each corresponding to a particular object (or the background), based on some user input. The proposed method reduces the interaction to the minimum, asking the user to just choose the object of interest by selecting a subregion inside it. The proposed approach does not attempt to estimate a specific model for a texture region, or an object, instead it tests for the homogeneity of a given feature pattern in a region: the features are the bands for each pixel in the image. In order to identify this homogeneity, the following assumptions about the image are made:

(a) the image contains a set of approximately homogeneous regions;
(b) spectral variables are also highly correlated that their dimension can be reduced without losing important information;
(c) the features between two neighbouring regions are distinguishable.

The first assumption requires images that present several regions with similar features, and we require a noise level that allows to distinguish the different regions. See [48,49,50] for a discussion on noise affecting data. Based on the second assumption we include a dimension reduction as a first processing step. For classification, in the lower dimensional space, we expect that data are well separated. Then, in the low dimensional space nearby points or points on the same structure (cluster or manifold) are likely to have the same label. In our case, nearby points are those pixels with similar features. Assumption (a) refers to a local property of the image: in an homogeneous region there is an high probability that a random walker remains in such region, whilst it is unlikely that it can reach a far but similar region. On the other hand, Assumption (b) refers to a global property of the image: similar regions may share similar spectral signature even if they are distant.

The classification results could be improved by using the contextual spatial information provided in the HS data in addition to the spectral information. In order to capture the spatial variations of the pixel features we consider closest fixed neighborhoods and a distance which involves all the pixels in these neighborhoods. Finally, the classification map is obtained by combining a random walk based model [51,52,53] with a direct label assignment: both are computed using this distance. The resulting energy changes the energy related to the random walker approach and improves the quality of the image classification. Moreover, the algorithm involves only the minimization of a quadratic function. We point out that the graph-based methods have been paid attention because of their solid mathematical background [47], relationship with sparseness properties, kernel methods, model visualization, and reliable results in many areas.

### 2.1. Regularized Linear Discriminant Analysis

When dealing with hyperspectral image analysis, one usually has a large number of spectral features *m* and a relative small number of training marked pixels $N_{T}$, divided in $n_{M}$ marked regions. A simple linear discriminant analysis (LDA) would then result in an ill-posed problem. In fact, from a theoretical point of view, one should deal with an infinite dimension Hilbert space to be linearly and compactly projected onto a finite dimensional one (see [54]). From a computational point of view, different strategies have been tested to overcome the ill-posedness of the problem. In [55] it is shown that an efficient version of the regularized LDA (RLDA) proposed in [56] performs better in segmenting hyperspectral images than support vector machine (SVM) classifiers and other LDA-based classifiers, such as penalized LDA, orthogonal LDA, and uncorrelated LDA.

The aim of RLDA is to find a projection matrix $\hat{G}\in R^{m\times l}$ to reduce the high-dimensional feature $x\in R^{m}$ to a lower dimensional vector $a={\hat{G}}^{⊤}x\in R^{l}$, l≪m. More precisely (see [55], Equation (10)),

(1) $$\hat{G}=\underset{G\in R^{m\times l}}{\arg\max}\left\{\mathrm{trace}\left({\left(G^{⊤}\left(S+\lambda I\right)G\right)}^{-1}G^{⊤}S_{b}G\right)\right\},$$

where *S* is the total scatter matrix, $S_{b}$ is the between-class variance matrix, *I* is the identity matrix, and λ is a regolarization parameter. Note that RLDA reduces to a classical LDA when λ=0.

The efficient Algorithm 1 for RLDA (see [55]) needs two singular values decompositions (SVD).
**Algorithm 1** Efficient RLDA given in [55]**Require:** λ≥0 **Ensure:** $\hat{G}$ as in (1) [U,D,V]←SVD(H)                ▹$H=UDV^{⊤}$ r←rank(H)=rank(D) $D_{s}\leftarrow D^{2}+\lambda I_{r}$ $\left[U_{b},D_{b},V_{b}\right]\leftarrow SVD\left(D_{s}^{-1/2}U^{⊤}H_{b}\right)$    ▹$D_{s}^{-1/2}U^{⊤}H_{b}=U_{b}D_{b}V_{b}^{⊤}$ $\hat{G}\leftarrow UD_{s}^{-1/2}U_{b}$


The first one computes the SVD of the normalized data matrix

$$H=\frac{1}{\sqrt{n_{L}}}\left[X_{T}-\mu 1^{⊤}\right]\in R^{m\times N_{T}},$$

where $X_{T}\in R^{m\times N_{T}}$ is the matrix data of the marked pixels and $\mu \in R^{m}$ is the feature mean vector of such data. The second SVD involves the normalized between-class data matrix

$$H_{b}=\frac{1}{\sqrt{N_{T}}}\left[\sqrt{n_{1}}\left(\mu_{1}-\mu \right),\sqrt{n_{2}}\left(\mu_{2}-\mu \right),\ldots ,\sqrt{n_{n_{M}}}\left(\mu_{n_{M}}-\mu \right)\right]\in R^{m\times n_{M}},$$

where $n_{j}$ (resp. $\mu_{j}$) denotes the sample size (res. the feature mean vector) for each marked region indexed by $j\in 1,\ldots ,n_{M}$. The challenging calculation of a ϵ-approximation of such SVDs may be faced with the new efficient randomized algorithms given in [57] (to this aim, Matlab introduced the function svdsketch since R2020b, that we decided to use).

Finally, the matrix $\hat{G}$, output of the Algorithm 1, is the matrix that projects the $n_{M}$ marked regions in a subspace that optimizes the Fisher score in a robust way [55]. We expect that this low-dimensional subspace will carry the relevant information to segment the entire figure. Hence, the original image $X_{0}\in R^{m\times n_{P}}$ is mapped to $Y={\hat{G}}^{⊤}X_{0}\in R^{l\times n_{P}}$.

### 2.2. A Spectral/Spatial Similarity Measure

After reducing the data, it is necessary to define an appropriate metric to establish the similarity between pixels in the mapped image *Y*. Each pixel in $y_{j}$ in *Y* has l≪m features (projected bands) that represent a vector in the linear space $R^{l}$. Instead, to focus on a pixel-wise similarity we fix a system of neighbourhoods with $N_{b}$ pixels, for each pixel, for example 8–neighborhoods, and a feature space, subset of $R^{l}$. Finally we collect all the entries of the feature vectors in a single vector $f_{j}\in R^{l\cdot N_{b}}$. We fix a distance $d_{l\cdot N_{b}}$ in the space $R^{l\cdot N_{b}}$. Then, for a couple of pixels $y_{j}$ and $y_{i}$, we define the similarity index $S\left(y_{j},y_{i}\right)={\left(d_{l\cdot N_{b}}\left(f_{j},f_{i}\right)+\varepsilon \right)}^{-1}$, ε≪1, that will be used in our experiments as a weight function, see (8) below. Moreover, the notion of similarity may be extended to a comparison between a pixel $y_{j}$ and a label $k\in \{1,\ldots ,n_{M}\}$. In fact, the region marked by label *k* is formed by pixels that have their features, from which we may extract a “centroid feature” ${\hat{f}}^{\left(k\right)}$, that best represents the labeled region. Then, for any pixel $y_{i}$ and any region *k*, the quantity

(2) $$S\left(i,k\right)={\left(d_{l\cdot N_{b}}\left(f_{i},{\hat{f}}^{\left(k\right)}\right)+\varepsilon \right)}^{-1}$$

will represent the similarity between the pixel *i* and the region *k*. This index can be interpreted as the first step of a *k*-means algorithm, whose starting centroids are computed with the user-marked regions as seeds.

This similarity, or the distance, could be used in a clustering algorithm because it represents a measure and/or comparison between pixels. Moreover, this distance allows to see the feature of the pixel in relation to its neighbors. This non-local method is inspired by recent works in signal analysis [58,59,60].

Moreover, using information from the patches instead of single pixels induces a smoothing effect [61] which may be useful in presence of noise. Indeed, consider a pixel $y_{j}$: denote with $f_{j}^{*}\in R^{l\cdot N_{b}}$ its corresponding feature vector and with $f_{j}∼f_{j}^{*}+N\left(0,\sigma^{2}I_{d}\right)$ its noisy version when Gaussian noise with zeros mean and covariance matrix $\sigma^{2}I_{d}$ is considered, being $I_{d}$ the identity matrix. Consider another pixel $y_{i}$ such that the intersection of the neighbourhoods of $y_{j}$ and $y_{i}$ is empty. We can give an estimation of the expected value $E\left[d_{l\cdot N_{b}}{\left(f_{j},f_{i}\right)}^{2}\right]$ of the square euclidean distance as

$$E\left[d_{l\cdot N_{b}}{\left(f_{j},f_{i}\right)}^{2}\right]={∥f_{j}^{*}-f_{i}^{*}∥}^{2}+2\cdot \left(l\cdot N_{b}\right)\sigma^{2}=d_{l\cdot N_{b}}{\left(f_{j}^{*},f_{i}^{*}\right)}^{2}+\left(2l\cdot N_{b}\right)\sigma^{2}$$

A similar estimation can be given when the noise affecting the pixels is not addictive but signal-dependent: for the case of Poisson noise $f_{j}∼\mathrm{Poiss}\left(f_{j}^{*}\right)$ one obtains

$$E\left[d_{l\cdot N_{b}}{\left(f_{j},f_{i}\right)}^{2}\right]=∥f_{j}^{*}-f_{i}^{*}{∥}^{2}+|f_{j}^{*}+f_{i}^{*}{|}_{1}=d_{l\cdot N_{b}}{\left(f_{j}^{*},f_{i}^{*}\right)}^{2}+{∥f_{j}^{*}+f_{i}^{*}∥}_{1}$$

where ${∥\cdot ∥}_{1}$ is the $\ell_{1}$ norm in $R^{l\cdot N_{b}}$ and $d_{l\cdot N_{b}}\left(f_{j}^{*},f_{i}^{*}\right)$ is the euclidean distance between the clean pixels. The above estimations are based on the fact that for a random variable *X* one has $E\left[X^{2}\right]=E{\left[X\right]}^{2}+\sigma^{2}\left(X\right)$, with $\sigma^{2}\left(X\right)$ the variance of *X*.

**Figure 1:**
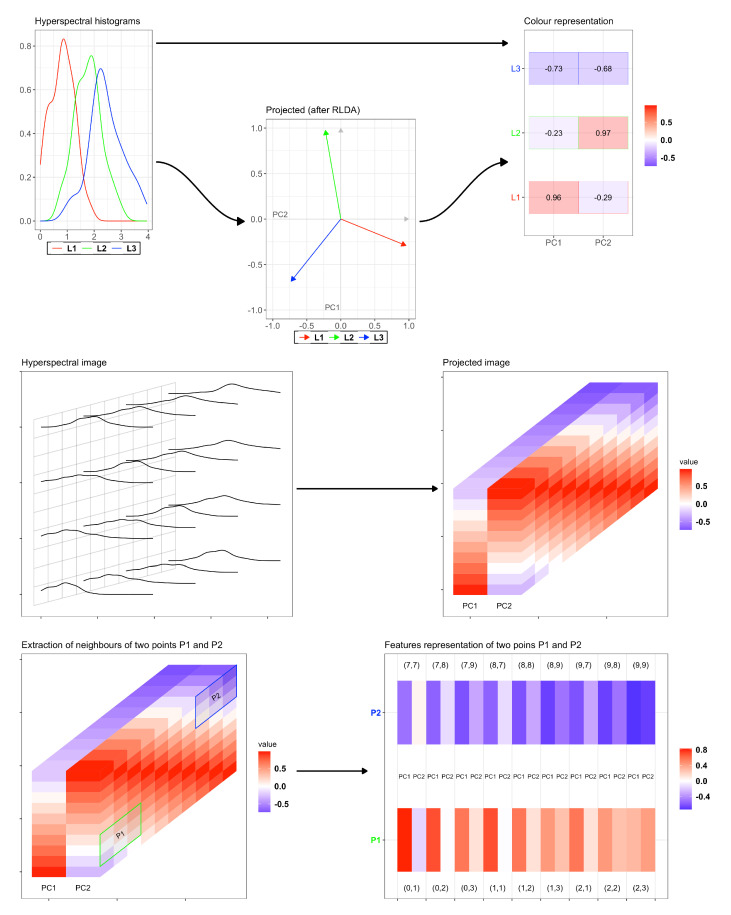
Visual representation of the RLDA algorithm and distance computation of the neighborhoods. First row: example with just 3 pixels and their hyperspectral distribution, reduced in their 2D principal components. Second row: RLDA applied to a larger image. Third row: extraction of the 8-neighborhoods of two pixels in each principal component.

Figure 1 depicts the while procedure for features compression and similarity computation. The first row is devoted to explain with a small example how the reduction is pursued. Consider just three pixels and their spectral distribution, i.e., the red, blue and green curves. The RLDA projection compresses each distribution in just a 2D vector: having at disposal these reduced vectors, one can compute the relative distance among these.

The second row depicts the procedure for a larger image: the grid on the left represents the pixels of the image, while the curves correspond to the spectral distributions for (some of) these pixels. Suppose that the dimension of the image is m×n×ℓ, where m×n is the dimension of the grid while *ℓ* refers to the number of recorded hyperspectral frequencies. The RDLA algorithm then compresses the distribution of each pixel in just two principal components (PC1 and PC2), providing a smaller data with size m×n×2. Consider now two pixels, namely $P_{1}$ and $P_{2}$, and their 8–neighborhoods in each component: in the previous notation, this hence amounts to have l=2 and $N_{b}=8$. Then, the vectors $f_{1}$ and $f_{2}$ that collect all the feature values of $P_{1}$ and $P_{2}$ and relative neighborhoods, respectively, belong to $R^{16}$: we can compute the similarity index as

$$S\left(P_{1},P_{2}\right)=\frac{1}{d_{16}\left(f_{1},f_{2}\right)+\varepsilon },\;\varepsilon \ll 1$$

with $d_{16}$ being a distance in $R^{16}$ (for example, the classical Euclidean distance).

### 2.3. The Random Walker Method

The random walker (RW) method (see [51]) is a segmentation method based on a “path distance”. It belongs to the classes of probabilistic methods, that allow to address several scientific tasks, such as optimization problems via Consensus-Based Optimization [62], Opinion Formation [63] or Particle Swarm Optimization [64].

More precisely, the RW method works as follows: in each image there are subregions of pixels which are marked with labels. Then, starting from a unmarked pixel, a random walk is performed along the whole image. Obviously, the subregions closer to the starting points are more likely to be first reached than the others. The random walk is then biased in order to promote those paths that involve similar pixels: a longer path with similar pixels may have a shorter “path distance” than a longer one that crosses non homogeneous regions.

More formally, the framework here involves an undirected graph G=(V,E), where $V=\{v_{i}|v_{i}\;\mathrm{is}\;a\;\mathrm{pixel}\;\mathrm{in}\;\mathrm{the}\;\mathrm{image}\}$ is the set of vertexes and *E* is the set of edges. Some pixels have been marked by the user, and will be denoted by $V_{m}$, the set of “marked vertices” in the graph. $V_{m}$ consists of $n_{M}$ marked regions. Define the set of labels for the marked vertices as a function $Q(v_{j})=k$, $\forall v_{j}\in V_{m}$. The other “unmarked vertices” of the image are denoted by $V_{u}$, so that $V=V_{m}⋃V_{u}$. The set of edges *E* will contain all the pairs of pixels which are neighbors in the original image, and $e_{ij}$ will denote the edge linking the vertexes $v_{i}$ and $v_{j}$.

The weight of an edge $e_{ij}$ encodes node similarity, and is represented by a function $\omega (v_{i},v_{j})$. If $d(v_{i},v_{j})$ represents some distance between two pixels, then common choices of ω are

$$\omega \left(v_{i},v_{j}\right)=e^{-\beta \;d{\left(v_{i},v_{j}\right)}^{2}}\;\mathrm{or}\;\omega \left(v_{i},v_{j}\right)=\frac{1}{\varepsilon +\sigma \;d(v_{i},v_{j})}$$

where the value of the parameters β,σ,ε>0 can be tuned accordingly. Starting form an unmarked vertex, a random walk is performed. At each step, when the walker is in the vertex $v_{i}$, the probability of reaching the neighbor $v_{j}$ depends on $\omega (v_{i},v_{j})$ proportionally: the highest values of the distance *d* will imply lower probabilities of reaching that neighbor. The algorithm then computes the probability of reaching any one of the labeled vertices belonging to one of the $n_{M}$ marked regions. Formally, for any vertex $v_{i}\in V_{u}$ and $k\in 1,\ldots ,n_{M}$, we denote by $x_{i}^{k}$ this probability. For a labeled node $v_{j}\in V_{m}$ that is associated to the label $Q\left(v_{j}\right)\in \left\{1,\ldots ,n_{M}\right\}$, we have $x_{j}^{k}=\delta_{i,Q(v_{i})}$. Image segmentation is then made on these probabilities, and the algorithm will tend to observe the weights of the image edges during the segmentation.

In [51], the computation of the probabilities $\left\{x_{i}^{k},i\in V_{u},k=1,\ldots ,n_{M}\right\}$ are calculated by solving a sparse linear system of equations, that involves the graph Laplacian matrix *L* associated to ω, that is defined as

$$L_{ij}=\left\{\begin{array}{cc} \sum_{v_{k}\;\mathrm{adjacent}\;\mathrm{to}\;v_{i}}\omega \left(v_{i},v_{k}\right), & \mathrm{if}\;i=j\\ -\omega (v_{i},v_{j}), & \mathrm{if}\;v_{i}\;\mathrm{and}\;v_{j}\;\mathrm{are}\;\mathrm{adjacent}\;\mathrm{nodes}\\ 0, & \mathrm{otherwise}. \end{array}\right.$$

In particular, for each label *k*, the probabilities $x^{k}={\left(x_{1}^{k},x_{2}^{k},\cdots ,x_{\left|V\right|}^{k}\right)}^{⊤}$ are found by solving the minimization of

(3) $$E\left(x^{k}\right)\;=\;\frac{1}{2}\sum_{(v_{i},v_{j})\in E}\;\omega \left(v_{i},v_{j}\right){\left(x_{i}^{k}-x_{j}^{k}\right)}^{2}\;=\;\frac{1}{2}{x^{k}}^{T}\;L\;x^{k}.$$

Note that, since L is positive semi-definite, the only critical points of *E* will be minima. In addition, since the corresponding continuous problem leads to the minimization of the Dirichlet integral via harmonic functions then the minimization problem (3) is also called combinatorial harmonic function (see [65]). The following problem

(4) $$x_{D}^{k}=\underset{x}{\mathrm{argmin}}E\left(x^{k}\right),\;k=1,\ldots ,n_{M}$$

is also called combinatorial Dirichlet problem.

We recall that $V=V_{m}⋃V_{u}$ and $V_{m}⋂V_{u}=\emptyset$. Without loss of generality, it is assumed that the nodes in L and x are ordered so that marked nodes comes first and unmarked nodes after. Therefore, for each $k\in \{1,\ldots ,n_{M}\}$, we may decompose the above formula (3) into

(5) $$E\left(x_{m}^{k},x_{u}^{k}\right)=\frac{1}{2}\left[{x_{m}^{k}}^{⊤},\;{x_{u}^{k}}^{⊤}\right]\left[\begin{array}{cc} L_{m} & B\\ B^{⊤} & L_{u} \end{array}\right]\left[\begin{array}{c} x_{m}^{k}\\ x_{u}^{k} \end{array}\right],$$

where $x_{m}^{k}$ and $x_{u}^{k}$ correspond to the probabilities of the labeled and unlabeled nodes, respectively, while B represents the anti-diagonal blocks of the Laplacian matrix. The problem could be interpreted as an interpolation of missing data (the unmarked points), while we have defined some (numerical) values for a subset of the vertices (our labeled nodes).

Omitting the superscript *k* for the ease of notation, the Equation (5) reads

$$E\left(x_{m},x_{u}\right)=\frac{1}{2}x_{m}^{⊤}L_{m}x_{m}+x_{u}^{⊤}B^{⊤}x_{m}+\frac{1}{2}x_{u}^{T}L_{u}x_{u}$$

and the unknowns are the entries of the vector $x_{u}$. Differentiating *E* with respect to $x_{u}$ and finding the critical point yields

(6) $$L_{u}x_{u}=-B^{⊤}x_{m}$$

which is a system of linear equations with $|V_{u}|$ unknowns. If every connected component contains at least a labeled vertex, then the Equation (6) is non-singular. Define $X_{u}=\left(x_{u}^{1},x_{u}^{2},\ldots ,x_{u}^{n_{M}}\right)$ and $X_{m}=\left(x_{m}^{1},x_{m}^{2},\ldots ,x_{m}^{n_{M}}\right)$, then the solution to the combinatorial Dirichlet problems (4) may be found by solving

(7) $$L_{u}X_{u}=-B^{⊤}X_{m}.$$

where $X_{m}$ is the labeling matrix with values in {0,1} such that $X_{m}1=1$ and $L_{u}$ is invertible. Thus, each unlabeled pixel $v_{i}$ gets $n_{M}$ probabilities $\left(x_{i}^{1},x_{i}^{2},\ldots ,x_{i}^{n_{M}}\right)$. Eventually, the label assigned to $v_{i}\in V_{u}$ corresponds to the index of maximum-by-rows in the solution of (7). For example, suppose that an image contains only $n_{M}=4$ marked regions. Consider just one pixel $v_{i}\in V_{u}$: the solution of (7) for this pixel reads as $\left(x_{i}^{1},x_{i}^{2},x_{i}^{3},x_{i}^{4}\right)=\left(0.15,0.5,0.25,0.1\right)$: this means that a random walker starting from $v_{i}$ has a probability of reaching the first region equal to 0.15, it has a probability of reaching the region labeled with k=2 of 0.5 and to reach the the region marked as k=3 or k=4 with probability of 0.25 or 0.1, respectively. This pixel is then labeled with as belonging to the second region, since a random walker is more likely attracted from that region. This approach is adopted also in [53].

Finally, in the next section we will test a combination of RW probabilities and similarity index, where the weights in Equation (3) are chosen as

(8) $$\omega \left(v_{i},v_{j}\right)=S\left(v_{i},v_{j}\right)=\frac{1}{d_{l\cdot N_{b}}\left(f_{i},f_{j}\right)+\varepsilon }\;,\;\varepsilon =10^{-3}.$$

### 2.4. A Local/Global Classification Method

Now, we consider the vertex labeling function, for simplicity we will consider labels represented by integers,

$$F_{L}:\;V\to S_{L}=\left\{1,\;2,\ldots ,\;n_{M}\right\},\;n_{M}\in N,n_{M}>1$$

which associates a label in a certain set to each vertex (pixel). Combining the RW approach, with the new distance defined above, and the new similarity measure, let $F_{L}$ as

(9) $$F_{L}\left(v_{i}\right)=\underset{k\in S_{L}}{\mathrm{argmax}}\;\left(S{\left(i,k\right)}^{\alpha }\;{\left(x_{i}^{k}\right)}^{1-\alpha }\right)$$

where S(i,k) is given in (2), $x_{i}^{k}$ are solutions of (7), and 1≥α≥0 is a parameter introduced for adding flexibility to the algorithm and to provide different weights to the two components of the labeling function. Due to the concavity of the logarithm function, and the positivity of S(i,k) and $x_{i}^{k}$, we can rewrite the labeling problem in an equivalent way as follows

(10) $$F_{L}\left(v_{i}\right)=\underset{k\in S_{L}}{\mathrm{argmax}}\left(\alpha \log\left(S\left(i,k\right)\right)+\left(1-\alpha \right)\log\left(x_{i}^{k}\right)\right).$$

The proposed method therefore can be summarized in Algorithm 2.
**Algorithm 2** Hyperspectral Random Walk by Similarity Index algorithm (HyperRaWaSI)Set the parameters α, λ, the neighbor system and the similarity function.Acquisition of user-marked pixels.Compute the projection of the original image $X_{0}$, $Y={\hat{G}}^{⊤}X_{0}$, where ${\hat{G}}^{⊤}$ is given by Algorithm 1.Compute the global similarity index S(i,k) for any $v_{i}\in V_{u}$ and $v_{k}\in V_{m}$.Solve system (7) for $V_{m}$, where the Laplacian matrix uses the index computed at step 3.Evaluate the labeling function as in (10) for the image $X_{0}$.

**Remark** **1.** *The two terms in the functional in (10) could be considered as a “fidelity term”, the αlog(S) part, and a regularizing term, the (1−α)log(x) part.*

**Remark** **2.** *The approach employed in our work is not simple post-processing: the similarity index with spatial information plays a role inside the classification decision. The optimization viewpoint depicted in Equation (10) raises a different framework from a post-processing one. Furthermore, the features distances affect at the same time the similarity between labeled and unlabeled pixels and on the construction of the graph for the part of the random walk.*

**Figure 2:**
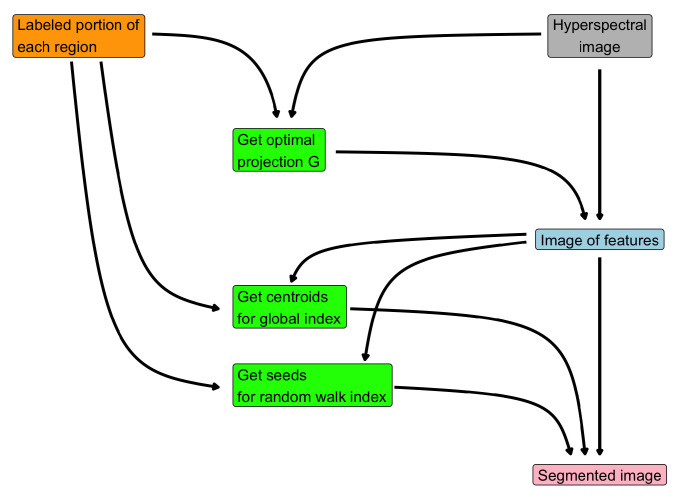
Flow chart of Algorithm 2.

Figure 2 provides a visual representation of the steps for Algorithm 2 and how they are related. The very first step is to acquire the user-marked region in the original image: this labels are the building blocks of the entire procedure, since

- they are employed to get the optimal projection of the hyperspectral image and hence the feature image;
- they are used to compute the centroids from the feature image;
- they represent the seeds for the random walker method.

The final segmentation is obtained by solving (10), which involves a convex combination of the similarity indexed image, obtained by the feature image and the centroids, and the output of the random walk method.

## 3. Results

In this section we present the numerical experiments done in order to assess the performance of the proposed algorithm. We used four different datasets, publicly available at http://www.ehu.eus/ccwintco/index.php?title=Hyperspectral_Remote_Sensing_Scenes (last visited: 17 October 2021):

- *University of Pavia*: this image was taken by the German Aerospace Agency (DLR) using the airborne ROSIS (Reflective Optics System Imaging Spectrometer) sensor. The spatial dimensions of the slices are 610×340 and the number of spectral band is 103: then the dataset size is 103× 207,400. The ground resolution is 1.3 m and the spectral gamma is 430–860 nm. The number *g* of ground truth labels is 9: Asphalt, Meadows, Gravel, Trees, Painted metal sheets, Bare soil, Bitumen, Self-blocking bricks, and Shadow.
- *Pavia Center*: this image refers to the center of the city of Pavia, but some samples in this dataset contain no information: the spatial size is 1096×1096 and it is then reduced to 1096×715. The considered spectral bands are 102, leading to a final size of 102× 783,640. The spectral bands lie in the interval 430–860 nm and the ground resolution is 1.3 m. The g=9 ground truth labels are Water, Trees, Asphalt, Self-Blocking Bricks, Bitumen, Tiles, Shadows, Meadows, Bare Soil.
- *KSC*: this image refers to the Kennedy Space Center, Florida (US) and it was acquired by the airborne AVIRIS (Airborne Visible/Infrared Imaging Spectrometer) NASA instrument. The spatial dimensions of the slices are 512×614 and the number of the spectral bands is 176: the size of the dataset is hence 176× 314,368. The ground resolution is 18 m and the spectral gamma is 400–2500 nm. The *g* ground truth labels are 13: Scrub, Willow swamp, CP hammock, CP/Oak, Slash pine, Oak/Broadleaf, Hardwood swamp, Graminoid marsh, Spartina marsh, Cattail marsh, Salt marsh, Mud flats, Water.
- *Indian Pines*: this image refers to the Indian Pines test site in North-Western Indiana, taken by AVIRIS sensor. The spatial dimensions are 145×145 and the employed bands are 220: The dataset size is hence 220× 21,025: the spectral gamma is 0.4–2.5 nm. The *g* ground truth labels are 16: Alfalfa, Corn-notill, Corn-mintill, Corn, Grass-pasture, Grass-trees, Grass-pasture-mowed, Hay-windrowed, Oats, Soybean-notill, Soybean mintill, Soybean-clean, Wheat, Woods, Building-grass-trees-drives, Stone-trees-drives.
- *Salinas HSI*: this image refers to an area including agricultural fields in the Salinas Valley, California, acquired again by AVIRIS sensor. The spatial size is 512×217 and the number of bands is 204: the dataset size is hence 204× 111,104 the ground resolution is 3.7 m, while the spectral gamma is 0.4–2.5 nm. The g=16 ground truth labels are: Broccoli gr. wds 1, Broccoli gr. wds, Fallow, Fallow rough plow, Fallow smooth, Stubble, Celery, Grapes untrained, Soil vineyard develop, Corn sen. gr. wds, Lettuce romaine 4 wk, Lettuce romaine 5 wk, Lettuce romaine 6 wk, Lettuce romaine 7 wk, Vineyard untrained, Vineyard vert. trellis.

The numerical experiments are organized as follows: the datasets *Indian Pines*, *Pavia University* and *Salinas HSI* are employed to assess the performance of the proposed algorithm, using suitable indexes and segmentation quality measurements. The datasets *Pavia Center* and *KSC* are used as real-world datasets: the labels contained in the ground truth are employed as seed for our segmentation technique.

All the experiments were carried on a laptop equipped with Linux 19.04, with an Intel(R) Core(TM) i5–8250U CPU (1.60 GHz), 16 GiB RAM memory (Intel, Santa Clara, CA, USA) and under MatLab R2020b environment (MathWorks, Natick, MA, USA). The code is available at https://github.com/AleBenfe/RaWaCs (last access: 17 October 2021).

### 3.1. Performance Measurements

This section makes use of the datasets *Indian Pines*, *Pavia University*, and *Salinas HSI*. As previously mentioned, the proposed method belongs to the class of semi-supervised techniques: the initial seeds, i.e., the user-marked regions, are in a lower number then the original ones, that are 16, 9, and 16 for *Indian Pines*, *Pavia University*, and *Salinas HSI*, respectively. We denote with $G_{t}$ the ground truth, which contains $N_{G}$ labels, and with ${\left\{g_{i}\right\}}_{i=1,\cdots ,g}$ the marked subsets. We select manually $n_{M}$ labels on the dataset: this will produce a segmentation $S_{t}$ result with $n_{M}$ marked regions ${\left\{s_{j}\right\}}_{j=1,\cdots ,n_{M}}$. In order to assess the performance when the ground truth contains a number *g* of labels greater than $n_{M}$, we make use of the following indexes.

- Rand Index (RI) [52]: this index measures if the two partitions of the image, namely $G_{t}$ and $S_{t}$, are coherent. For any couple of pixels $\left(p_{1},p_{2}\right)$ in the ground truth $G_{t}$, the RI measures the coherence between the partitions: it checks if $p_{1}$ and $p_{2}$ belongs to the same subset $g\in G_{t}$ and at the same time they belongs to the same subset $s\in S_{t}$. It checks also if $p_{1}$ and $p_{2}$ belongs to the two different subsets $g_{1},g_{2}\in G_{t}$ and at the same time they belongs to two different subsets $s_{1},s_{2}\in S_{t}$. Denote with $n_{s}$ the number of couples that belongs to the same subset in $G_{t}$ and that belong to the same subset in $S_{t}$, while denote with $n_{d}$ the number of couples that do not belong to the same subset in $G_{t}$ and that do not belong to the same subset in $S_{t}$, then
  RI=ns+ndnG2
  where $n_{G}$ is the total number of pixels labeled in $G_{t}$ and nG2 refers to the number of all possible couples. The best performances are obtained when the RI is close to 1.
- Overall Accuracy (OA): for each true label $i=1,\ldots ,n_{G}$, we compute
  $$\hat{ı}=\arg\max_{j=1,\cdots ,n}\left\{\#\left(g_{i}\cap s_{j}\right)\right\}.$$
  This classifies the label *i* as belonging to the region of $s_{\hat{ı}}\subseteq S_{t}$, marked with $\hat{ı}$. The Overall Accuracy is defined as
  $$OA=\frac{\sum_{i=1}^{N_{g}}\#\left(g_{i}\cap s_{\hat{ı}}\right)}{\sum_{i=1}^{g}\#\left(g_{i}\right)}=\frac{\sum_{i=1}^{N_{g}}\max_{j=1,\cdots ,n}\left\{\#\left(g_{i}\cap s_{j}\right)\right\}}{n_{G}},$$
  which measures the average number of pixels of the ground truth $G_{t}$ that are truly classified together. The best performances are obtained when the OA is close to 1.

**Figure 3:**
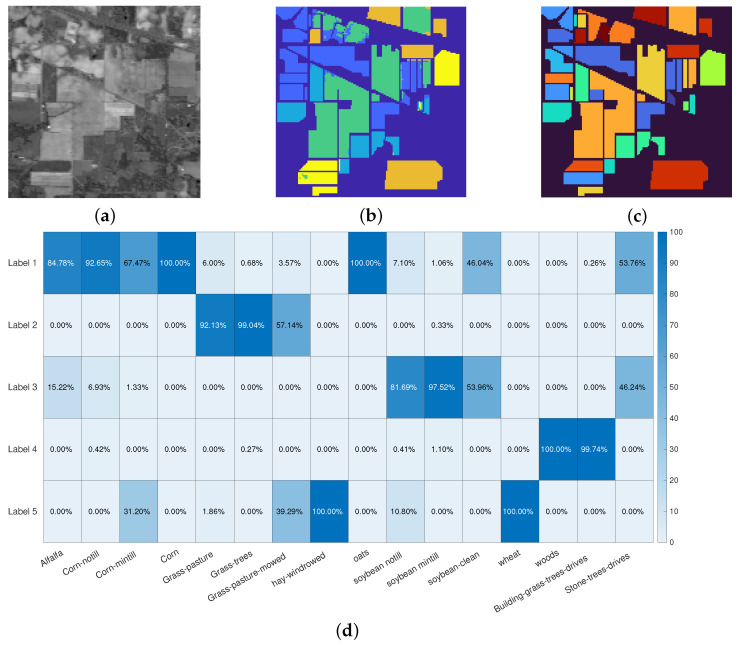
Indian Pines segmentation results. (**a**): false gray scale image. (**b**): segmentation result. (**c**): true labels. (**d**): accuracy over the classes. The colormaps in (**b**,**c**) are different because the segmentation process takes into account 5 labels, while the ground truth contains 16 labels. Each region in the ground truth falls almost entirely in one of the 5 manually selected labels.

The first experiment refers to the *Indian Pines* dataset: the original image is depicted in Figure 3a, the segmentation result in Figure 3b whilst the ground truth labels are in Figure 3b. These results are obtained by setting λ=0.01 and α=0.8 in Algorithm 2. The Rand Index for this particular experiment is 0.8505, while the OA is 0.9029: this means that each user-marked label contains the most part of a region marked in the ground truth. This result is clearly shown in Figure 3d: the element in (i,j) position shows the percentage of the pixels in ground truth label *j* which are marked as belonging to the user-marked label *i*.

**Figure 4:**
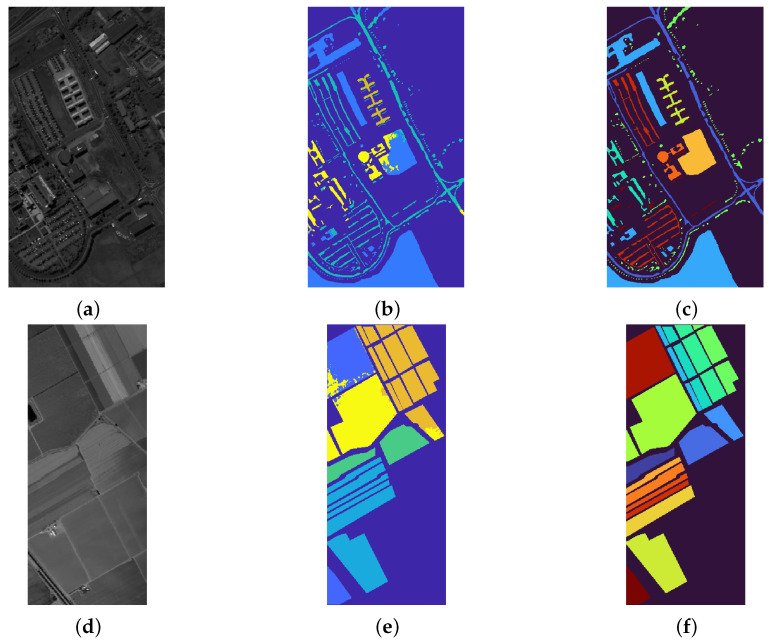
(**a**–**c**): false grayscale image, segmentation result and ground truth labels for the *Pavia University* dataset. (**d**–**f**): false grayscale image, segmentation result and ground truth labels for the *Salinas HSI* dataset. The former experiments achieves an Overall Accuracy of 0.93, whilst the latter achieves an OA of 0.9696.

The *Pavia University* and *Salinas HSI* datasets present very similar results, with the same setting for the parameters (α=0.8,λ=0.01): the segmentation on the former provides a RI of 0.8060 and a OA of 0.9393, and the proposed procedure reaches a reliable performance on the latter too, with RI = 0.8704 and OA = 0.9696. These experiments show that the proposed procedure is able to properly cluster several regions, according to common hyperspectral properties, even when the initial seeds are selected via a visual inspection of some of the bands presented in the dataset. Figure 4 presents the results for the *Pavia University* and *Salinas HSI* datasets.

Table 1 collects the computational times of the entire procedure (excluding the marking process) for each image: even for very large datasets such as *Pavia University* the proposed algorithm is very fast. We employed the MatLab function svdsketch which implements the RLDA procedure depicted in Algorithm 1.

**Figure 5:**
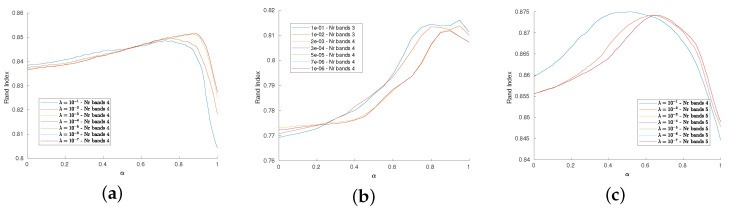
(**a**–**c**): Dependence of the RI wrt α for several values of λ, from 1 to $10^{-6}$ for *Indian Pines*, *Pavia University* and *Salinas HSI* datasets, respectively. The RI remains high, there is a peak around α=0.85 in the former cases, while the *Salinas HSI* datasets achieve its best performance for α=0.65 when λ is lower than 1. The number of bands obtained by the dimensionality reduction is stable wrt λ.

We now assess the dependence of the performance on the parameters α and λ: the plots in Figure 5 depict the behavior of the Rand Index with respect to α in correspondence of several choice for λ, namely from λ=1 to $\lambda =10^{-6}$. These plots show that the proposed procedure is quite stable with respect to this settings, showing an increasing of the performance around α=0.8. Moreover, the dimensionality reduction seems to be independent on the choice of λ. As already noted in [46], the best performances are achieved when α≠0 and α≠1, meaning that the coupling (in this case, via a convex combination) of the RW approach and of the similarity index is the optimal strategy with respect to selecting only one of the two methods.

### 3.2. Comparison with State–of–the–Art Methods

**Figure 6:**
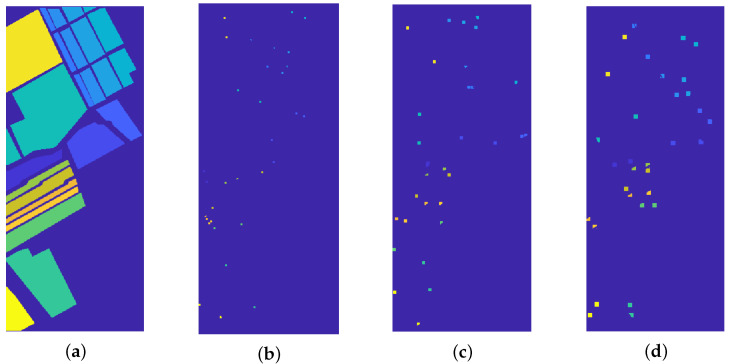
(**a**) Ground truth labels. (**b**–**d**) Random seeds for the proposed procedure, chosen among the ground truth mask. From left to right: square seeds of dimension 3, 5, and 7 pixels. The squares are clipped in order to refer to the correct region.

This section is devoted to compare the proposed strategy with state-of-the-art methods, namely K-MBC [21], FCM [66], FDPC [67], and GWEEN [68], when the true number of clusters is known. The seeds employed for our method are randomly chosen in the ground truth mask: for each marked region, two small squares with size 7 pixels are selected. See Figure 6 for a visual explanation of this process and for example of different square size.

Table 2 presents the comparison between our method and the other ones using the Overall Accuracy and the Purity indexes as evaluations. The Purity index measures how much pure a cluster is, i.e., its tendency to contain a sole class. The performance indexes regarding the other methods are taken from ([21], Tables 4 and 5). The setting for the proposed procedure are α=0.93,0.92 and α=0.91 for *Salinas*, *Pavia*, and *KSC* datasets, respectively, while λ=0.1 for all 3 datasets.

The proposed procedure overcomes all the methods used in the comparison, both on overall accuracy and on purity indexes.

We test the performance of the strategy with respect to the size and shape of the user-marked regions. We consider the *salinas* and *Pavia* datasets, and we employ as user-marked regions two randomly chosen squares with size 3, 5, and 7 (see Figure 6). The results are depicted in Table 3.

As one expects, the larger the marking size, the higher the performances: even with a very small marking (3×3 square), the overall accuracy is above 80% and the purity is greater than 90% in the case of *salinas* dataset, meaning that the recognized clusters contain mostly one class.

### 3.3. Segmentation as Atlas

In this section the *Pavia Center* and *KSC* datasets are segmented using the ground truth labels as seeds. The idea beyond this strategy relies on the concept of *atlas* (see [69]): an atlas is a collection of objects whose labels are known. The strategy consists in merging the images of the atlas with the one which has to be segmented, hence the resulting image in this way contains a labeled part (from the atlas) which allows the classification of the rest of the data.

We apply this strategy to the *Pavia Center* and *KSC* datasets: we adopted this approach since the subsets labeled in the ground truth data are low in number in both cases, despite the dimension of the images.

**Figure 7:**
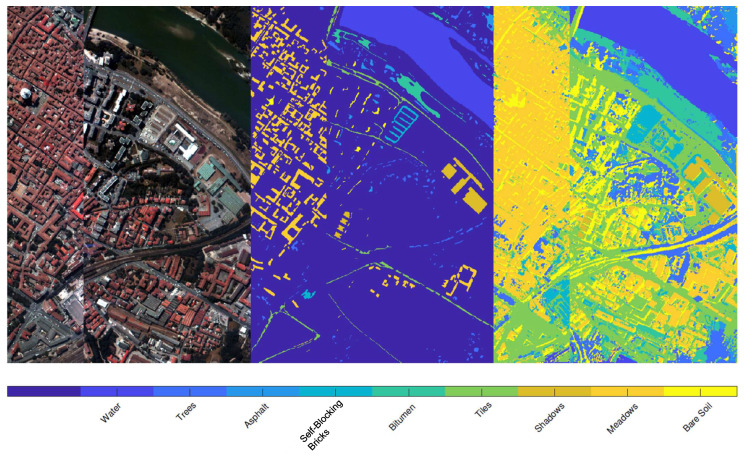
Result of the segmentation of *Pavia Center* using the ground truth labels as atlas. From left to right: image of the landscape in false colors, labels of the ground truth, final result. The latter panel shows that all the roofs are remarkably recognized, as the shadows they project on the ground. The vegetation is segmented with a very high level of precision. We reported the labels as reported in the database we used for these experiments: there are clearly some errors, since some classes (such as Shadows, Meadows and Bare Soils) refers to objects that are not the ones described by these labels.

Figure 7 refers to the *Pavia center* dataset: in the left panel a colored image of the landscape is depicted, while the central panel shows the labels contained in the ground truth. The strategy consists then of using these labeled regions as an atlas, providing exact information for the classification, without requiring user intervention. The final result, obtained with α=0.9 and λ=0.2 is given in the last panel of Figure 7: a visual inspection of the achieved segmentation suggests that the recognition of the various regions is remarkable and precise. Indeed, all the red roofs, together with their projected shadow, are recognized in a remarkable way. The trees are well segmented even if few markings of them are contained in the atlas. Note that there is an abrupt interruption inside the image, as previously mentioned in the description at the beginning of this section: despite this discontinuity, the proposed procedure does not suffer from the presence of such issue in the dataset, providing a segmentation that can take into account that the image is not continuous.

**Figure 8:**
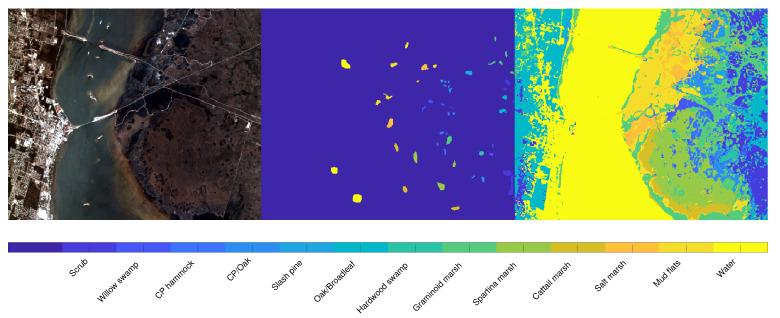
Segmentation results on the *KSC* dataset. From left to right: false color image, ground truth labels employed as seeds, and segmentation result. On the bottom of the images the legend associates the color to the labels.

**Figure 9:**
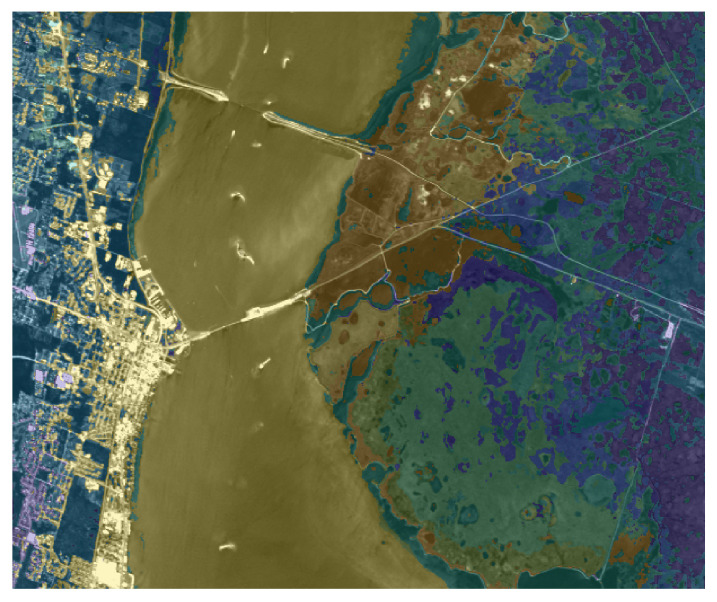
Overlay between the segmentation and original image (in grayscale) for the *KSC* dataset.

We apply the same strategy (using the ground truth as an atlas) to the *KSC* dataset, where the main aim is to segment the different type of vegetation. Figure 8 presents the visual representation of the segmentation result, obtained by setting α=0.97 and $\lambda =10^{-4}$. The right part of the image present a reliable segmentation, since the different parts of terrain and vegetation are well recognized. On the other hand, the segmentation result on the left part classifies some buildings as part of vegetation or water, since the ground truth does not present any label referring to human constructions, such as buildings, bridges or streets. Figure 9 is devoted to showing the reason why some buildings are included in the Water label: indeed, the river contains several white parts which the segmentation process correctly includes in the Water label. On the other hand, there are several buildings that present a spectral signature (both in the visible and in the infrared region) very similar to these white regions inside the river. The picture shown in Figure 9 also better exposes the reliable performance on the right part of the image.

We finally report that the computational times for the segmentation of *Pavia Center* and *KSC* datasets, which present both large dimensions: 102× 783,640 the former and 176× 314,368 the latter. In both cases, the computational time is close to being negligible with respect to the size (see Table 4).

## 4. Conclusions

In this work we proposed a new spectral/spatial semi-automatic classification method coupling a Regularized Linear Discriminant analysis for dimension reduction, a suitable similarity index, and a random walker approach. The final computational problem involves a new energy function obtained by a new definition of similarity/distance between pixel using a reduced features space and pixels in a neighborhood. The experimental results showed that the proposed approach is very robust with respect to the presence of noise and with good accuracy. In our approach we have the flexibility of a system of different neighborhoods for the calculation of the distance between pixels and in the construction of the graph corresponding to a given image.

Regarding the hyper-parameters λ and α, we observed that in the regularization process the choice of the λ parameter does not seem to influence the numerical results even varying it by several orders of magnitude. The RW part encourages the selection of convex zones, consequently changing the α parameter in Equation (10) can give more weight to these sub-regions in the final classification. In a future paper we will consider appropriate learning methods for the optimal choice of these parameters for some classes of images.

From the computational point of view, the most expensive steps concern the calculation of the new distance between pixels and the preliminary reduction step (the computation of the projection operator ${\hat{G}}^{⊤}$, see Algorithm 1). However, we observe that these operations can be performed efficiently in parallel, for example with an appropriate implementation through the use of GPUs. Moreover, we adopted a fast state-of-the-art SVD algorithm. The computation of the probabilities of RW requires the numerical solution of linear systems which may be large, but sparse and well structured at the same time, consequently, efficient algorithms can be used. Then, our method is reliable and efficient also for high-definition images.

We point out that it is possible to introduce some set of suitable atlas through the projection matrix $\hat{G}$, see Algorithm 1. We plan to explore the combination of atlas and our classifier using an adaptive approach in order to learn better weights to be employed in the features distance. Moreover, we will consider the possibility to replace some or all the labeled pixels with atlases well adapted to the specific image. Preliminary results were shown and discussed in Section 3.3. Further comparisons will be made with other semi-automatic methods, identifying suitable quality measures of the segmentation obtained.

## Figures and Tables

**Table 1:** Computational time in seconds.

Dataset	Size	Time
*Pavia University*	103× 207,400	1.08
*Indian Pines*	220× 21,025	0.13
*Salinas HSI*	204× 111,104	0.45

**Table 2:** Comparison between methods.

	*Salinas*		*Pavia*		KSC	
Method	O.A.	Purity	O.A.	Purity	O.A.	Purity
FCM	54.6	0.63	41.9	0.47	52.5	0.56
FDPC	62.2	0.71	44.2	0.54	47.6	0.64
GWEEN	65.3	0.77	47.9	0.62	49.8	0.69
K–MBC	76.5	0.93	65.9	0.91	58.2	0.74
HyperRaWaSI	**93.8**	**0.94**	**91.1**	**0.90**	**97.8**	**0.98**

**Table 3:** Performance behavior with respect to the label size.

	*Salinas*		*Pavia*	
Size	O.A.	Purity	O.A.	Purity
3	89.9	0.92	80.1	0.80
5	92.7	0.94	82.7	0.81
7	93.8	0.94	91.1	0.90

**Table 4:** Computational time in seconds.

Dataset	Size	Time
*Pavia University*	102× 783,640	5.24
*KSC*	176× 314,368	3.31

## Data Availability

On 3 December 2021, the code for the MatLab implementation of RaWaCs is available at https://github.com/AleBenfe/RaWaCs. Publicly available datasets were analyzed in this study. On 17 October 2021, data were found at: http://www.ehu.eus/ccwintco/index.php?title=Hyperspectral_Remote_Sensing_Scenes.
